# Trends in Gout Incidence and Management during the COVID-19 Pandemic: A Nationwide Study in England via OpenSAFELY

**DOI:** 10.1016/S2665-9913(23)00206-0

**Published:** 2023-08-31

**Authors:** Mark D Russell, Jon Massey, Edward Roddy, Brian MacKenna, Seb Bacon, Ben Goldacre, Colm D Andrews, George Hickman, Amir Mehrkar, Arti Mahto, Andrew I Rutherford, Samir Patel, Maryam A Adas, Edward Alveyn, Deepak Nagra, Katie Bechman, Joanna M Ledingham, Joanna Hudson, Sam Norton, Andrew P Cope, James B Galloway

**Affiliations:** Centre for Rheumatic Diseases, King’s College London, SE5 9RJ, UK; Bennett Institute for Applied Data Science, Nuffield Department of Primary Care Health Sciences, University of Oxford, Oxford, OX2 6GG, UK; School of Medicine, Keele University, Keele, ST5 5BG, UK; Bennett Institute for Applied Data Science, Nuffield Department of Primary Care Health Sciences, University of Oxford, Oxford, OX2 6GG, UK; Bennett Institute for Applied Data Science, Nuffield Department of Primary Care Health Sciences, University of Oxford, Oxford, OX2 6GG, UK; Bennett Institute for Applied Data Science, Nuffield Department of Primary Care Health Sciences, University of Oxford, Oxford, OX2 6GG, UK; Bennett Institute for Applied Data Science, Nuffield Department of Primary Care Health Sciences, University of Oxford, Oxford, OX2 6GG, UK; Bennett Institute for Applied Data Science, Nuffield Department of Primary Care Health Sciences, University of Oxford, Oxford, OX2 6GG, UK; Bennett Institute for Applied Data Science, Nuffield Department of Primary Care Health Sciences, University of Oxford, Oxford, OX2 6GG, UK; Department of Rheumatology, King’s College Hospital NHS Foundation Trust, London, SE5 9RS, UK; Department of Rheumatology, King’s College Hospital NHS Foundation Trust, London, SE5 9RS, UK; Centre for Rheumatic Diseases, King’s College London, SE5 9RS, UK; Centre for Rheumatic Diseases, King’s College London, SE5 9RS, UK; Centre for Rheumatic Diseases, King’s College London, SE5 9RS, UK; Centre for Rheumatic Diseases, King’s College London, SE5 9RS, UK; Centre for Rheumatic Diseases, King’s College London, SE5 9RJ, UK; Rheumatology Department, Portsmouth Hospitals University NHS Trust, Portsmouth, PO6 3LY, UK; Department of Psychology, Health Psychology Section, Institute of Psychiatry, Psychology & Neuroscience, King’s College London, UK; Centre for Rheumatic Diseases, King’s College London, SE5 9RJ, UK; Centre for Rheumatic Diseases, King’s College London, SE5 9RJ, UK; Centre for Rheumatic Diseases, King’s College London, SE5 9RJ, UK

## Abstract

**Background:**

Gout is the most prevalent inflammatory arthritis, yet one of the worst managed. Our objective was to assess how the COVID-19 pandemic impacted on incidence and care quality for people with gout in England.

**Methods:**

With the approval of NHS England, we conducted a population-level cohort study using primary care and hospital data for 17.9 million adults via the OpenSAFELY platform. We analysed the following outcomes between 1 March 2015 and 28 February 2023: 1) incidence and prevalence of recorded gout diagnoses; 2) incidence of gout hospitalisations; 3) initiation of urate-lowering therapy (ULT); and 4) serum urate target attainment.

**Findings:**

From 17,865,145 adults, there were 246,695 incident gout diagnoses. The mean age of diagnosed patients was 61.3 years (SD 16.2), 66,265 (26.9%) were female, and 189,035 (90.9%) of 208,050 with available ethnicity data were White. Newly recorded gout diagnoses decreased by 31.0% in the year beginning March 2020, compared with the preceding year (1.23 vs. 1.78 diagnoses per 1,000 adults). Gout prevalence was 3.07% in 2015/16 and 3.21% in 2022/23. Gout hospitalisations decreased by 30.1% in the year commencing March 2020, relative to the preceding year (9.58 vs. 13.7 admissions per 100,000 adults). Of 228,095 people with incident gout and available follow-up, 66,560 (29.2%) were prescribed ULT within 6 months. Of 65,305 ULT initiators with available follow-up, 16,790 (25.7%) attained a urate ≤360 micromol/L within 6 months of ULT initiation. In interrupted time-series analyses, ULT prescribing improved modestly during the pandemic, relative to pre-pandemic, while urate target attainment was similar.

**Interpretation:**

Using gout as an exemplar disease, we demonstrated the complexity of how healthcare was impacted during the pandemic. We observed a reduction in gout diagnoses but no impact on treatment metrics. Importantly, we showed how country-wide, routinely-collected data can be used to map disease epidemiology and monitor care quality.

**Funding:**

None

## Introduction

Gout is the most prevalent inflammatory arthritis worldwide, but one of the worst managed. ^1^ Guidelines recommend discussing and/or offering preventative urate-lowering therapies (ULT; e.g. allopurinol) to all patients with gout, followed by titration of ULT dosing until serum urate targets are achieved. ^2,3^ Despite this, studies from prior to the COVID-19 pandemic had shown persistently low uptake of ULT and poor attainment of urate targets. ^1,4,5^

The COVID-19 pandemic has had an enormous impact on service delivery throughout healthcare systems worldwide, with abrupt changes to healthcare utilisation, re-deployment of staff, and a rapid transition to virtual consultations. ^6–8^ The extent to which this has affected care for people with long-term conditions, such as gout, is not understood.

The OpenSAFELY data analytics platform provides a unique opportunity to address this question. Through OpenSAFELY, pseudonymised electronic health records (EHR) for up to 99% of England’s population can be analysed in a highly-secure environment in near real-time. In a recent proof-of-concept study, a 20% reduction in autoimmune inflammatory arthritis diagnoses was observed during the first year of the pandemic in England; however, for people who sought medical attention, the impact of the pandemic on the delivery of care for diagnoses such as rheumatoid arthritis (RA) was less marked than might have been expected. ^9^

Our objective was to assess how the COVID-19 pandemic has impacted on diagnostic incidence and care quality for people with gout in England.

## Methods

### Study design and data source

We performed a population-level, observational cohort study using EHR data via the OpenSAFELY platform. Due to data availability, we piloted our approach in OpenSAFELY-TPP, which contains data for 23 million people, including 17.9 million adults (approximately 40% of the population of England). OpenSAFELY-TPP is representative of England’s population in terms of age, sex, Index of Multiple Deprivation (IMD), ethnicity and causes of death. ^10^ Primary care records managed by the GP software provider, TPP, were linked to NHS Secondary Uses Service data through OpenSAFELY.

### Incident and prevalent case definitions

The study period was from 1 March 2015 to 28 February 2023. Incident gout diagnoses were defined as people aged 18-110 years, registered with TPP practices in England for at least 12 months, who had index diagnostic codes for incident gout (see appendix p5 for codelists). At least 12 months of continuous registration prior to diagnosis was required for incident diagnoses, to ensure only index diagnoses were captured. People with incident gout codes who had received prescriptions for ULT more than 30 days before diagnosis were deemed not to be incident diagnoses.

The incidence of gout was defined as the number of newly recorded gout diagnoses within the study population during each study year (from 1 March to 28 February). The study population was defined as people registered with TPP practices for at least 12 months at the mid-point of each study year (1 September); this assumed individuals were registered for the full study year. We calculated the point prevalence of gout by dividing the number of people with prevalent diagnostic codes for gout (see appendix p5 for codelists) at a fixed time point - chosen as the mid-point of each study year (1 September) - by the number of people currently registered with TPP practices at that time point. No age or sex standardisation of incidence or prevalence was performed due to the relatively short study period, with only minimal differences in age or sex distribution observed over this time period (Table 1).

### Incidence of gout hospitalisations

Linked data on hospitalisations were available from 1 April 2016 to 31 March 2022. The incidence of gout hospitalisations was defined as the number of hospitalisations with primary admission diagnoses of gout (ICD10 code: M10) within the study population during each year (from 1 April to 31 March). The study population was defined as the number of people registered with TPP practices at the mid-point of each study year.

### ULT initiation and serum urate target attainment

National Institute for Health and Care Excellence (NICE) guidelines recommend discussing the option of ULT with all people diagnosed with gout, followed by titration of ULT dosing until a serum urate ≤360 micromol/L (≤6mg/dL) is achieved. ^3^ For people with incident gout who had at least 6 months of available follow-up after diagnosis, we reported the proportion who received a prescription for ULT (allopurinol or febuxostat) within 6 months of diagnosis. Primary care prescriptions were captured, but prescriptions dispensed by hospital pharmacies were not.

For people with incident gout prescribed ULT within 6 months of diagnosis who had at least 6 months of available follow-up after initiating ULT, we reported the proportion who attained a serum urate ≤360 micromol/L within 6 months of ULT initiation.

### Statistical methods

Baseline sociodemographic characteristics and comorbidities were described without inferential statistics for people with incident gout (presented overall and by diagnosis year) and for the reference population (at 1 March 2019). Details of comorbidity definitions and codelists are included within the appendix, p5.

Interrupted time-series analyses (ITSA) were performed to estimate the impact of the pandemic on the proportion of incident gout patients, averaged by month, who were: i) prescribed ULT within 6 months of diagnosis; ii) prescribed ULT within 6 months of diagnosis and attained a serum urate ≤360 micromol/L within 6 months of ULT initiation. Trends were compared before and after the first COVID-19 lockdown in England (March 2020) using single-group ITSA. ^11^ Autocorrelation between observation periods was accounted for using Newey-West standard errors with 5 lags. ^11^ Outcomes were also presented by region of England (categorised into the 9 Nomenclature of Territorial Units for Statistics (NUTS) Level 1 regions^12^) using horizontal bar charts.

Python 3.8 was used for data management and Stata 16 for statistical analyses. All code for data management and analysis, as well as codelists, are shared openly for review and re-use under MIT open license (https://github.com/opensafely/gout). As our analyses were primarily descriptive, no correction for multiple hypothesis testing was performed. For statistical disclosure control, frequency counts were rounded to the nearest 5 and non-zero counts below 8 were redacted.

### Study approval and ethics

Approval to undertake this study under the remit of service evaluation was obtained from King’s College Hospital NHS Foundation Trust. No further ethical approval was required as per UK Health Research Authority guidance. This study was supported by Dr Joanna Ledingham as senior sponsor. An information governance statement is included at the end of this manuscript.

### Role of funding source

No study funders were involved in the study design, collection, analysis or interpretation of data, in the writing of the report, or in the decision to submit for publication.

### Patient and public involvement

This analysis relies on the use of large volumes of patient data. Ensuring patient, professional and public trust is therefore of critical importance. Maintaining trust requires being transparent about the way OpenSAFELY works, and ensuring patient voices are represented in the design of research, analysis of the findings, and considering the implications. For transparency purposes, OpenSAFELY have developed a public website (https://opensafely.org/) which provides a detailed description of the platform in language suitable for a lay audience; they have participated in two citizen juries exploring public trust in OpenSAFELY; ^13^ they are currently co-developing an explainer video; they have ‘expert by experience’ patient representation on the OpenSAFELY Oversight Board; they have partnered with Understanding Patient Data to produce lay explainers on the importance of large datasets for research; they have presented at a number of online public engagement events to key communities; and more. To ensure the patient voice is represented, OpenSAFELY are working closely with appropriate medical research charities.

## Results

### Baseline characteristics

From a reference population of 17.9 million adults, there were 246,695 incident gout diagnoses between 1 March 2015 and 28 February 2023. A study flowchart is shown in Supplementary Figure S1. Relative to the reference population, people with incident gout were older (mean age 61.3 vs. 49.7 years; standard deviation 16.2 vs. 18.7 years, respectively), more likely to be male (73.1% vs. 49.8%), and have more comorbidities including obesity (45.4% vs. 27.9%), hypertension (47.0% vs. 21.4%), diabetes mellitus (18.5% vs. 9.6%), chronic cardiac disease (19.9% vs. 6.8%), chronic kidney disease (24.0% vs. 6.5%), and diuretic use (26.1% vs. 5.9%) (Table 1).

### Incidence and prevalence

**Figure 1:**
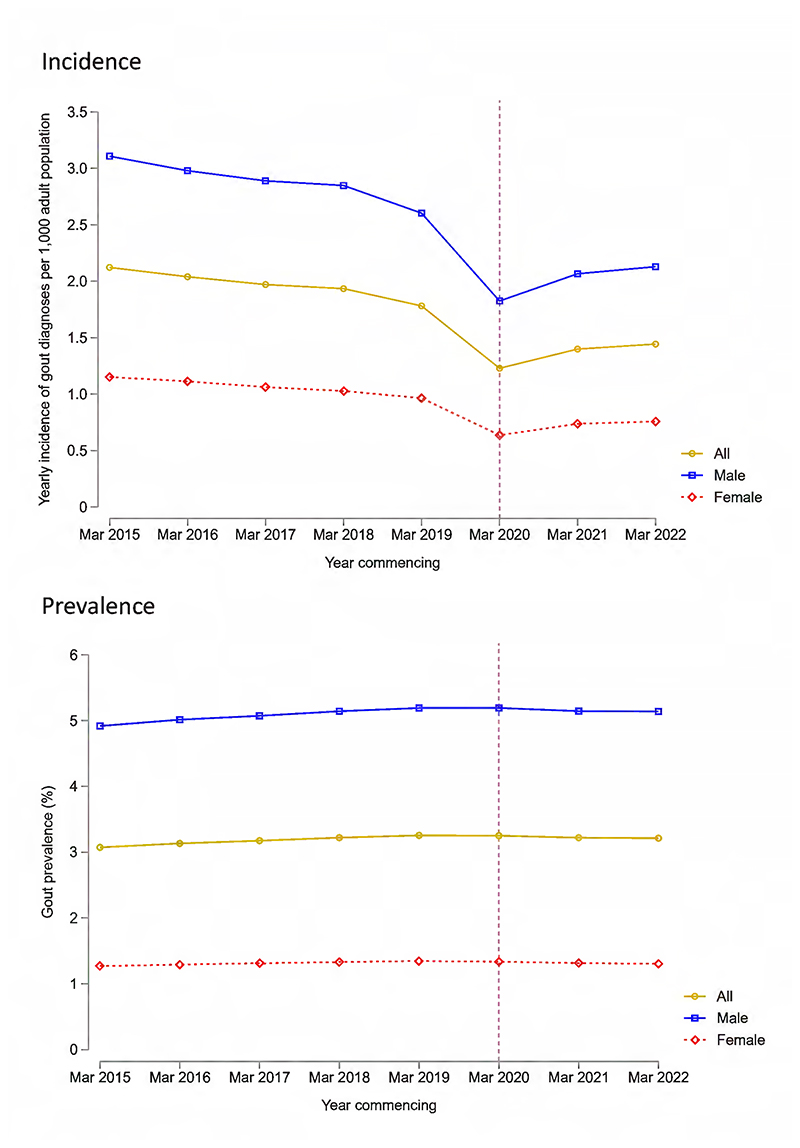
Incidence (top panel) and prevalence (bottom panel) of gout diagnoses recorded in primary care in England between 1 March 2015 and 28 February 2023. Incidence and prevalence are shown overall and separated by male and female sex. The vertical dashed line corresponds to the onset of the first COVID-19 lockdown in England (March 2020).

The incidence of newly recorded gout diagnoses decreased from 2.12 per 1,000 adults in 2015/16 to 1.78 per 1,000 adults in 2019/20 (Figure 1 and Supplementary Figure S2). A marked decrease in recorded gout diagnoses was observed in the year beginning March 2020, compared with the year preceding the pandemic, corresponding to a 31.0% decrease in incidence (from 1.78 to 1.23 diagnoses per 1,000 adults). This was driven primarily by a 39.0% decrease in recorded diagnoses between February 2020 and April 2020 (from 2,475 to 1,510 monthly diagnoses, respectively). The incidence of recorded gout diagnoses increased in the years commencing March 2021 and March 2022 (1.40 and 1.44 diagnoses per 1,000 adults, respectively), but remained below pre-pandemic incidence.

Gout prevalence remained relatively stable over the study period, at 3.07% of adults in 2015, 3.25% in 2019, and 3.21% in 2022 (Figure 1). Hospitalisations with primary admission diagnoses of gout increased from 12.2 per 100,000 adults in 2016/17 to 13.7 per 100,000 adults in 2019/20, before decreasing by 30.1% during the first year of the pandemic, to 9.58 admissions per 100,000 adults (Supplementary Figure S3). A modest increase in admissions was observed in the year commencing March 2021 (10.7 admissions per 100,000 adults), but this remained before pre-pandemic levels.

### Trends in urate-lowering therapy

**Figure 2:**
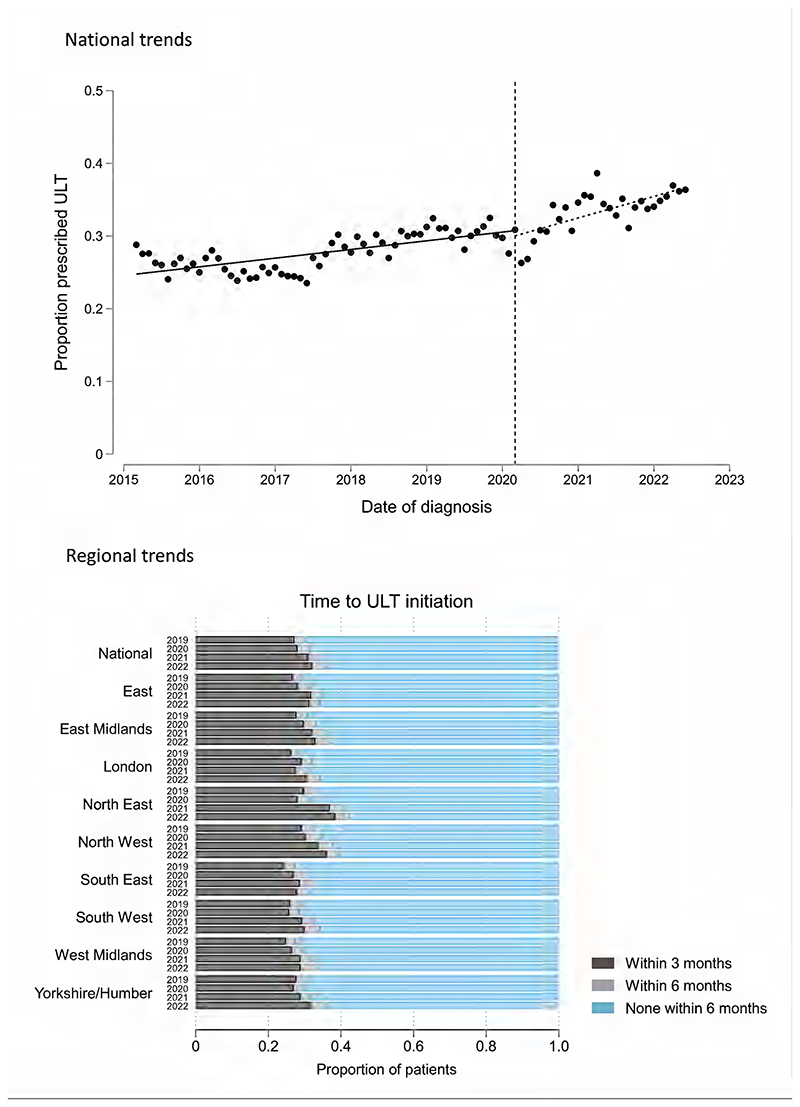
Trends in the proportion of patients with incident gout who were initiated on ULT. In the top panel, an interrupted time series analysis shows national trends in the mean monthly proportion of patients who initiated ULT within 6 months of diagnosis. The vertical dashed line corresponds to the onset of the first COVID-19 lockdown in England (March 2020). The bottom panel shows the proportion of patients who were prescribed ULT within 3, 6, or >6 months of diagnosis, separated by region of England and by year (March 2019/20; March 2020/21; March 2021/22; March 2022/23).

Of 246,695 new gout diagnoses during the study period, 228,095 (92.5%) had at least 6 months of available follow-up, 66,560 (29.2%) of whom were prescribed ULT within 6 months of diagnosis (65,680/206,890 [31.8%] within 12 months of diagnosis). In ITSA models, modest improvements in ULT initiation were observed over the study period (Figure 2). Small, statistically significant improvements in ULT prescribing trends were seen after March 2020, relative to pre-pandemic trends: trend pre-March 2020: 1.19% improvement per year (95% CI 0.69 to 1.70); trend post-March 2020: 2.96% improvement per year (95% CI 1.58 to 4.35); difference in trends: 1.77% improvement per year (95% CI 0.23 to 3.30; p=0.025). Improvements in ULT initiation during the pandemic were observed throughout most regions of England, albeit to varying degrees (Figure 2).

### Trends in serum urate target attainment

**Figure 3:**
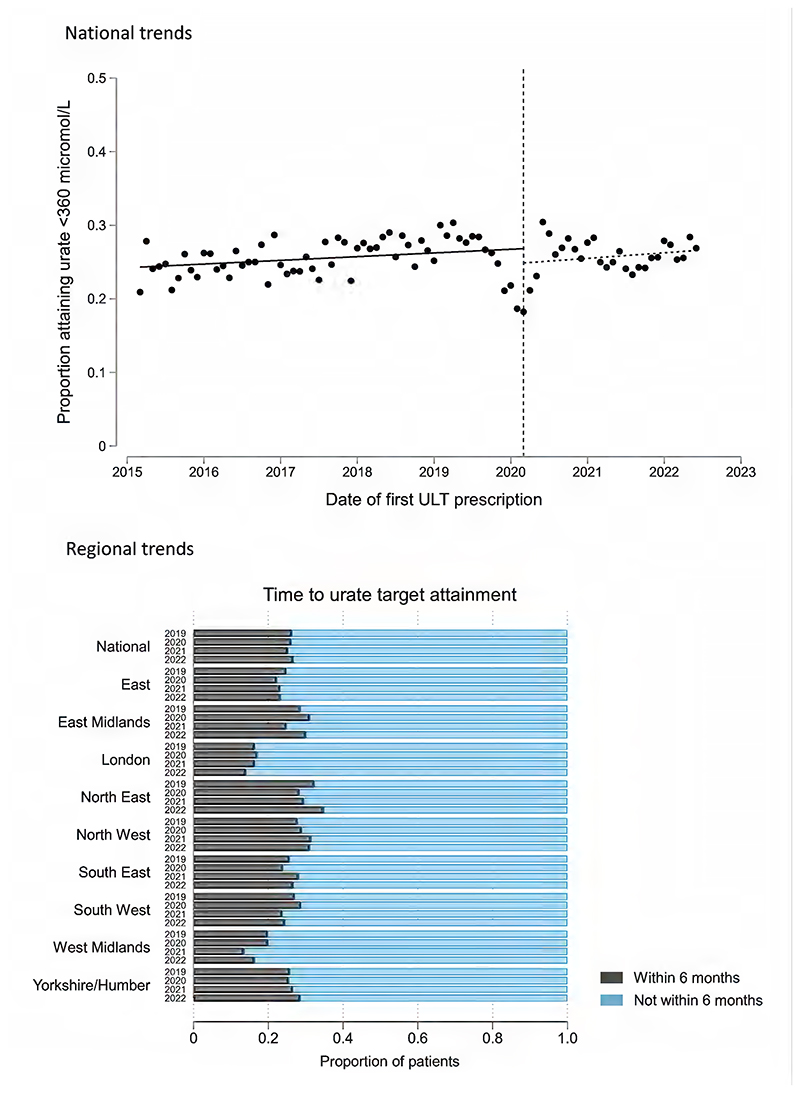
Trends in the proportion of incident gout patients who attained serum urate levels ≤360 micromol/L within 6 months of initiating ULT. In the top panel, an interrupted time series analysis shows national trends in the mean monthly proportion of patients who attained target within 6 months of ULT initiation. The vertical dashed line corresponds to the onset of the first COVID-19 lockdown in England (March 2020). The bottom panel shows the proportion of incident gout patients who attained a urate ≤360 micromol/L within 6 months of ULT initiation, separated by region of England and by year (March 2019/20; March 2020/21; March 2021/22; March 2022/23).

Of 66,560 patients with incident gout who initiated ULT within 6 months of diagnosis, 65,305 (98.1%) had at least 6 months of available follow-up after ULT initiation. 36,245/65,305 (55.5%) patients had at least one serum urate level performed within 6 months of initiating ULT, while 12,990/65,305 (19.9%) had two or more urate levels performed. 16,790/65,305 (25.7%) attained a serum urate ≤360 micromol/L within 6 months of ULT initiation (18,170/58,455 [31.1%] within 12 months). Urate target attainment remained relatively stable over the study period, aside from a temporary decrease in attainment for people initiating ULT in late 2019 and early 2020 (nadir of 18.2% in March 2020), before recovering by June 2020 (Figure 3). Overall, there were no significant differences in urate target attainment trends before and after the onset of the pandemic: trend pre-March 2020: 0.50% improvement per year (95% CI -0.31 to 1.31); trend post-March 2020: 0.75% improvement per year (95% CI -1.18 to 2.69); difference in trends: 0.25% improvement per year (95% CI -2.21 to 2.71; p=0.84). Urate target attainment varied considerably throughout England during the pandemic, with the lowest attainment seen in London (185/1,155; 16.0%) and highest attainment seen in North-East England (555/1,800; 30.8%) (Figure 3).

### Characteristics of people presenting before and after pandemic onset

Differences in patients presenting with new gout diagnoses during each year of the pandemic, relative to before the pandemic, were investigated (Table 1). The age, sex, ethnicity, and sociodemographic composition of patients presenting during the pandemic were comparable to patients presenting before the pandemic. Proportionately fewer patients presenting with gout during the pandemic had comorbid hypertension, chronic kidney disease or diuretic use, relative to before the pandemic. The proportion of patients with tophi at diagnosis was comparable before and after the onset of the pandemic, as was early flare burden. Serum urate levels at diagnosis were also comparable in patients presenting before vs. during the pandemic.

## Discussion

In this study, we used the OpenSAFELY platform to demonstrate a marked reduction in recorded gout diagnoses during the COVID-19 pandemic in England. No increase in gout diagnoses above pre-pandemic levels has been observed as of 3 years after the pandemic’s onset, suggesting a substantial burden of undiagnosed disease. For people presenting with new gout diagnoses during the pandemic, small improvements in ULT initiation were seen, relative to pre-pandemic trends, while trends in serum urate target attainment were comparable. Irrespective of the pandemic, ULT initiation and urate target attainment remain far below an acceptable standard.

This study demonstrates the potential to transform monitoring of chronic diseases using routinely-collected health data. Unlike existing national audits (e.g. the National Early Inflammatory Arthritis Audit in England and Wales), ^14^ the use of routinely-collected health data in Trusted Research Environments obviates the need for manual data entry by clinicians, increases case ascertainment, and reduces the potential for bias. ^9,15^ Rates of ULT initiation and urate target attainment in our study were comparable to studies utilising other data sources (e.g. CPRD), supporting the validity of our approach. ^5,16^ In contrast to these other data sources, however, analyses using OpenSAFELY can be updated in near real-time and do not require any sharing of potentially identifiable patient data, minimising the risk of sensitive data disclosure.

The 40% decrease in incident gout diagnoses observed in the early months of the pandemic is comparable to what has been described for autoimmune inflammatory arthritis (IA) diagnoses, such as rheumatoid arthritis (RA). ^9^ This highlights the wide-ranging impact of the pandemic on both primary care and secondary care-led rheumatological conditions, with service provision disrupted across many parts of the country due to redeployment of staff. National data show that 10% fewer primary care appointments occurred in England between April 2020/2021, relative to the preceding year, which is likely to have contributed to some but not all of the observed reduction in recorded gout diagnoses during the pandemic. ^17^ Similarly, our finding of a 30% reduction in gout hospitalisations during the first year of the pandemic needs to be considered in the wider context of a 16% reduction in all-cause emergency admissions in England between April 2020/2021, relative to April 2019/2020. ^18^ In addition to the marked reduction in recorded gout diagnoses observed during the pandemic, we also observed a background decrease in gout incidence over the full study period. This supports the findings of a recent observational study, utilising CPRD, that reported a decreasing incidence of gout that predated the COVID-19 pandemic, with a potential link to changes in alcohol intake and dietary modification over time. ^16^

As was reported for autoimmune IA diagnoses, the absence of a rebound increase in recorded gout diagnoses above pre-pandemic levels suggests many people remain undiagnosed as a consequence of the pandemic. ^9^ It remains to be seen the degree to which this represents people who have yet to seek medical attention (e.g. due to altered health-seeking behaviour) or people yet to be diagnosed due to ongoing system-wide pressures. Gout is characterised by episodic flares early in the disease course, with intercritical periods that can last several months or years. As such, it is possible that patients who did not seek medical attention for index gout flares during the pandemic may not yet have experienced further flares and/or re-presented to primary care; this may have contributed to the absence of a rebound increase in gout diagnoses over the relatively short study period.

Our findings highlight the remarkable adaptation of the health service to the pandemic; for example, in being able to deliver modest improvements in ULT initiation despite unprecedented pressures. This reflects what has been reported for other IA diagnoses, including RA, where time to first rheumatology assessment and DMARD initiation were comparable or better than before the pandemic. ^9^ The rapid transition to virtual consultations during the pandemic may have favoured conditions such as gout, for which remote titration of urate-lowering therapies is possible. Despite this, absolute levels of ULT initiation and urate target attainment remained sub-optimal at the end of the study period (at 34% and 29%, respectively), while only 20% of patients had more than one urate level performed within 6 months of initiating ULT. This demonstrates the pressing need for strategies to encourage uptake of treat-to-target ULT.

In addition to benchmarking national standards of care, our data highlight marked regional variation in gout care. Urate target attainment in certain regions of England (e.g. North East England) was close to double that of other regions (e.g. London). Regional disparities in care were evident before the pandemic and, in some cases, have become more pronounced since the pandemic. Further research incorporating qualitative methodology is needed to better understand the reasons behind such disparities. This could help tailor the implementation of strategies towards addressing regional facilitators and barriers to better care, which, in turn, could be monitored over time using electronic dashboards based upon near real-time updates of these data.

In contrast to other IA diagnoses, where some markers of disease severity (e.g. DAS28) captured by specialist clinics are not currently available for analysis in OpenSAFELY, we were able explore differences in patients presenting with gout during vs. before the pandemic. We hypothesised that patients presenting during the pandemic were more likely to be those with more severe disease, particularly in the context of increased weight gain and alcohol consumption during the pandemic. ^19,20^ Our findings did not support this hypothesis. The proportion of patients who had tophi at baseline (a marker of disease severity) was similar during and before the pandemic, as was the proportion of patients who experienced recurrent flares after diagnosis (a marker of disease burden). Serum urate levels at baseline were also comparable. Of note, proportionately fewer patients presenting with gout during the pandemic had comorbidities such as chronic kidney disease. This could represent altered health-seeking behaviour in such patients; for example, in response to government recommendations for high-risk patients to stay at home (‘shield’) during the pandemic. ^21^

Our study had limitations. Although our estimates of gout incidence and prevalence are in line with other studies utilising EHR data, ^4,16^ there is a potential for diagnostic misclassification inherent to studies using coded health data, which can lead to overestimates of incidence and prevalence. With EHR studies, one must also acknowledge the challenges in determining whether observed differences in diagnostic incidence over time represent true changes in underlying disease incidence or changes in the recording of diagnoses. While the marked decrease in gout diagnoses observed during the pandemic is likely to primarily reflect delays in presentation and the recording of diagnoses, further research is needed to determine whether longer-term trends reflect true decreases in disease incidence. As our analyses centred on gout diagnoses coded in primary care in England, they may not be representative of secondary care gout management during the pandemic or generalisable to other countries. Additionally, we could only capture primary care-issued prescriptions for ULT in OpenSAFELY, not secondary care-issued prescriptions; ^22^ however, as the majority of patients with gout are managed in primary care, this is unlikely to have meaningfully altered our findings.

When interpreting the observed changes in ULT prescribing, it is important to acknowledge changes in guideline recommendations that have occurred over time, which may have influenced prescribing behaviour. In the 2017 BSR gout management guidelines, it is recommended that all patients with gout should be offered ULT, including those presenting with their first flare. ^2^ In the NICE gout guideline, introduced in 2022, there is a recommendation to discuss the option of ULT with all patients with gout, but there is no specific recommendation to offer ULT unless additional factors are present (e.g. multiple flares, tophi or CKD). ^3^ If the NICE criteria were applied over the full study period, then the proportion of patients who should have been offered ULT and were prescribed ULT would have been relatively higher. Similarly, we could not account for patient preference in our analyses; for example, patients who were offered ULT by their clinician but declined to start it. Finally, we were unable to describe other important aspects of gout care in our analyses, such as patient-reported outcomes and the provision of disease education.

In conclusion, we showed that newly recorded gout diagnoses decreased by a third during the first year of the pandemic, with no rebound increase in incidence observed as of early 2023. For patients who presented with incident gout, ULT initiation improved modestly during the pandemic, while urate target attainment was comparable to before the pandemic. Despite this, absolute levels of ULT initiation and urate target attainment remain below an acceptable standard. Importantly, this study demonstrates the potential for routinely-captured health data to revolutionise the monitoring of chronic diseases at both national and regional levels.

## Supplementary Material

Supplementary

## Data Availability

All data were linked, stored and analysed securely within the OpenSAFELY platform (https://opensafely.org/). Data include pseudonymised data such as coded diagnoses, medications and physiological parameters. No free text data are included. All code for data management and analysis, as well as codelists, are shared openly for review and re-use under MIT open license (https://github.com/opensafely/gout). Detailed pseudonymised patient data are potentially re-identifiable and therefore not shared. Access to the underlying identifiable and potentially re-identifiable pseudonymised electronic health record data is tightly governed by various legislative and regulatory frameworks and is restricted by best practice. The data in OpenSAFELY are drawn from general practice data across England where TPP is the data processor. TPP developers (Chris Bates, Jonathan Cockburn, John Parry, Frank Hester, and Sam Harper) initiate an automated process to create pseudonymised records in the core OpenSAFELY database, which are copies of key structured data tables in the identifiable records. These are linked onto key external data resources that have also been pseudonymised via SHA-512 one-way hashing of NHS numbers using a shared salt. Bennett Institute for Applied Data Science developers and PIs (Ben Goldacre, Liam Smeeth, Jon Massey, Seb Bacon, Alex J Walker, William Hulme, Helen J Curtis, David Evans, Peter Inglesby, Simon Davy, George Hickman, Krishnan Bhaskaran, and Christopher T Rentsch) hold contracts with NHS England and have access to the OpenSAFELY pseudonymised data tables as needed to develop the OpenSAFELY tools. These tools in turn enable researchers with OpenSAFELY Data Access Agreements to write and execute code for data management and data analysis without direct access to the underlying raw pseudonymised patient data, and to review the outputs of this code. All code for the full data management pipeline—from raw data to completed results for this analysis—and for the OpenSAFELY platform as a whole is available for review at https://github.com/OpenSAFELY. The data management and analysis code for this paper was led by MDR and JBG.

## Research in context

### Evidence before this study

We performed a systematic literature search to identify population-level, observational cohort studies that compared the incidence, prevalence and management of gout before and during the COVID-19 pandemic. We searched PubMed for articles published from database inception to 6 June 2023 using the terms “incidence”, “prevalence”, “management”, “treatment”, “gout” and “COVID”. No studies with data beyond 2021 were identified. A study performed in the UK, with data to 2021, reported a 23% reduction in incident gout cases between 2019 and 2021, while reductions in serum urate target attainment were observed for patients initiating ULT in 2019 and 2020. A study in South Korea reported no significant changes in gout incidence between 2016 and 2020.

### Added value of this study

We used the OpenSAFELY platform of 17.9 million adults in England to analyse changes in gout incidence, prevalence, management and hospitalisations between 1 March 2015 and 28 February 2023. On a background of decreasing gout incidence and stable prevalence, we showed that newly recorded gout diagnoses decreased by 31% (from 1.78 to 1.23 diagnoses per 1,000 adults) in the first year of the pandemic, relative to pre-pandemic, before recovering partially. People presenting with gout during the pandemic did not have more severe disease than those presenting pre-pandemic, but did have proportionately fewer comorbidities. ULT initiation trends improved modestly for people presenting with incident gout during the pandemic, relative to before the pandemic, while urate target attainment trends were similar; however, absolute levels of ULT initiation and urate target attainment remained sub-optimal as of February 2023.

### Implications of all the available evidence

The incidence of recorded gout diagnoses decreased markedly during the pandemic. No rebound increase in incidence has been observed as of early 2023, which suggests that many patients with gout are yet to be diagnosed as a result of the pandemic. For patients who were diagnosed with gout during the pandemic, ULT initiation improved relative to before the pandemic. However, absolute levels of ULT initiation and urate target attainment remain below an acceptable standard. Importantly, our study demonstrates the potential for routinely-captured health data to transform how we monitor disease epidemiology and care quality.
