# Supplementary material for: Trends in Gout Incidence and Management during the COVID-19 Pandemic: A Nationwide Study in England via OpenSAFELY
Source: Lancet Rheumatol. Author manuscript; Available in PMC 2024 Aug 15. (PMC7616140; doi:10.1016/S2665-9913(23)00206-0)
Supplement: Supplementary [file EMS197227-supplement-Supplementary.docx]

**Contents**

[**Supplementary Figure S1**. Flow diagram of study populations utilised in our analyses. 2](#_Toc142476433)

[**Supplementary Figure S2.** Monthly incidence of gout diagnoses newly recorded in primary care in England between 1 March 2015 and 28 February 2023. 3](#_Toc142476434)

[**Supplementary Figure S3.** Yearly incidence of hospitalisations with primary admission diagnoses of gout in England between 1 April 2016 and 31 March 2022. 4](#_Toc142476435)

[**Supplementary Data:** Diagnostic codelists and comorbidity information 5](#_Toc142476436)

[**Information Governance Statement** 6](#_Toc142476437)

[**References** 7](#_Toc142476438)

## **Supplementary Figure S1**. Flow diagram of study populations utilised in our analyses.


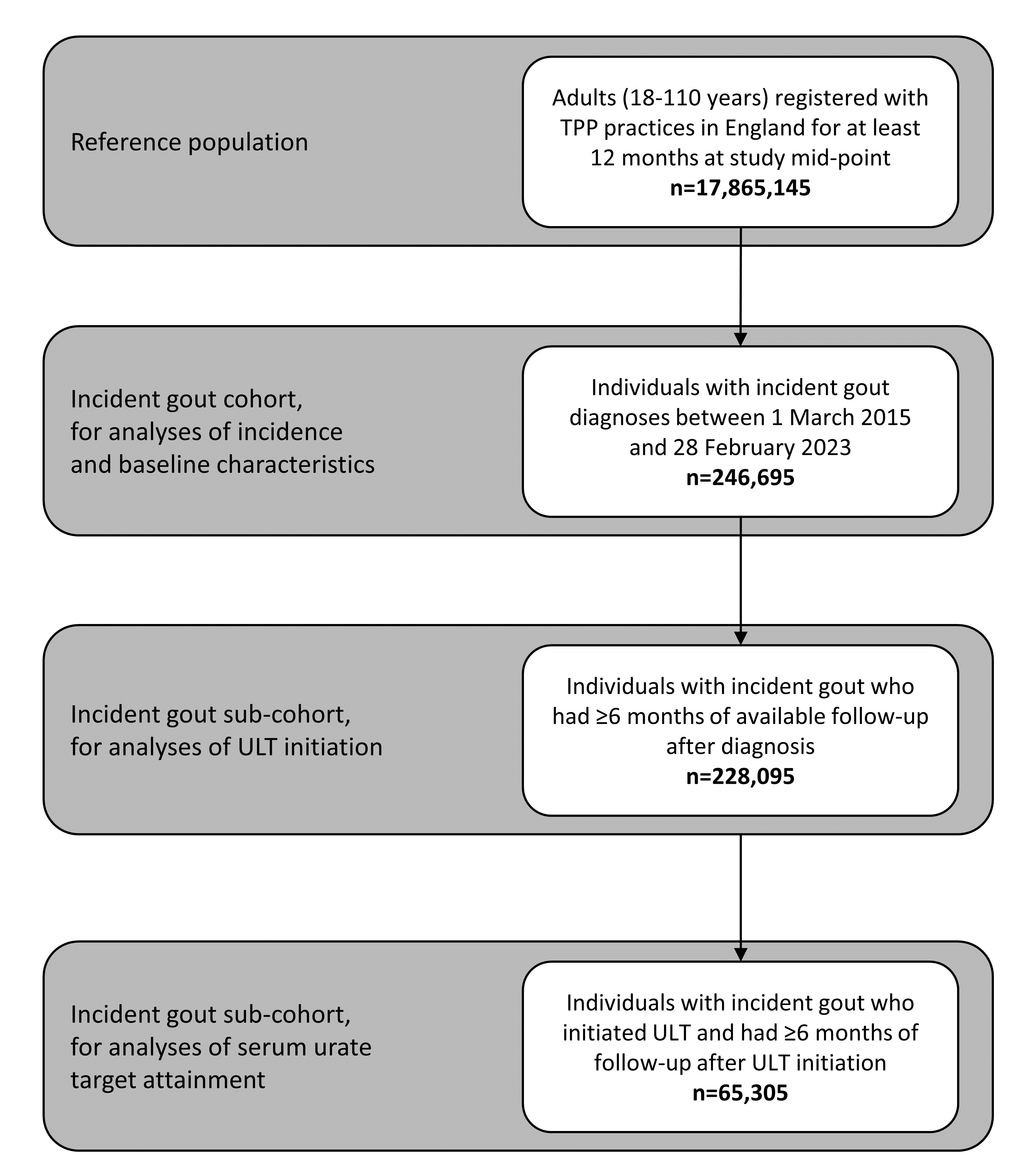


ULT: urate-lowering therapy; TPP: primary care software provider.

## **Supplementary Figure S2.** Monthly incidence of gout diagnoses newly recorded in primary care in England between 1 March 2015 and 28 February 2023.


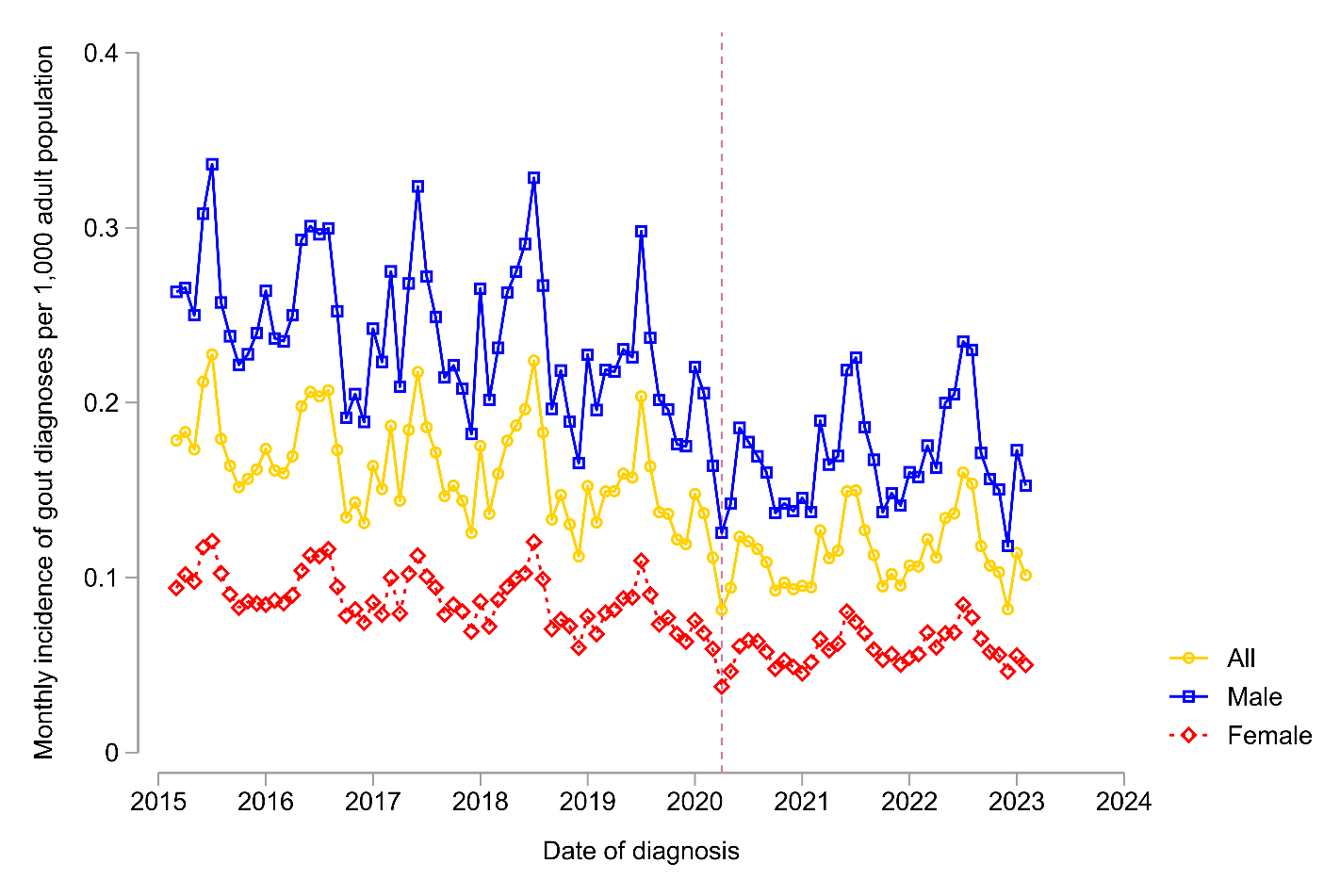


Incidence is shown overall and separated by male and female sex. The vertical dashed line corresponds to the onset of the first COVID-19 lockdown in England (March 2020).

## **Supplementary Figure S3.** Yearly incidence of hospitalisations with primary admission diagnoses of gout in England between 1 April 2016 and 31 March 2022.


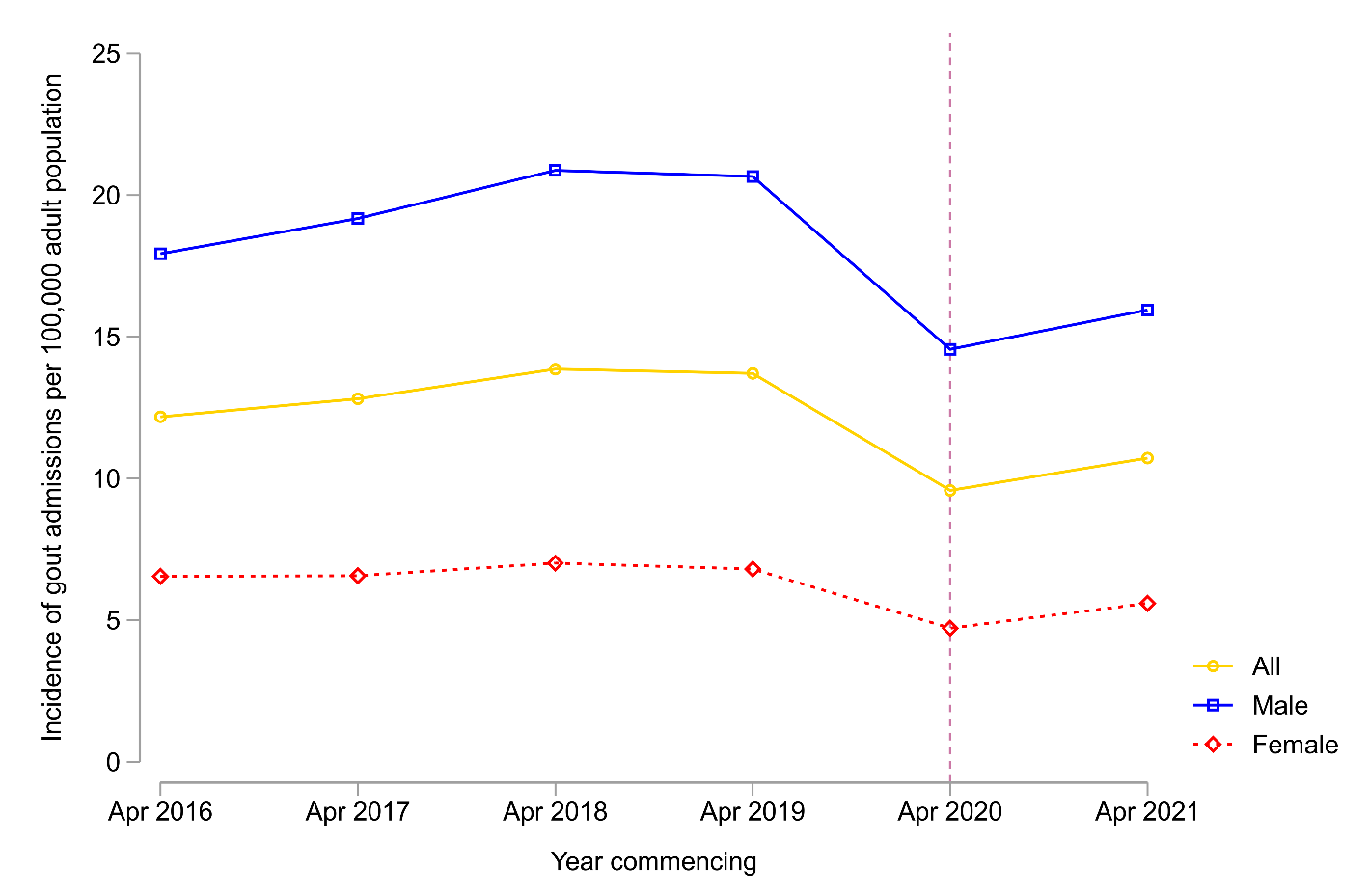


Incidence and prevalence are shown overall and separated by male and female sex. The vertical dashed line corresponds to the onset of the first COVID-19 lockdown in England (March 2020).

## **Supplementary Data:** Diagnostic codelists and comorbidity information

**Diagnostic codelists**

- Incident gout diagnoses: <https://www.opencodelists.org/codelist/user/markdrussell/gout/7a2a1f9e/>
- Prevalent gout diagnoses: <https://www.opencodelists.org/codelist/user/markdrussell/gout-prevalent/048d1131/>
- Gout admissions: <https://www.opencodelists.org/codelist/user/markdrussell/gout-admissions/07a7df6d/>
- Gout flare: <https://www.opencodelists.org/codelist/user/markdrussell/gout-flaresattacks/5334de55/>
- Tophaceous gout: <https://www.opencodelists.org/codelist/user/markdrussell/gouty-tophi/41df05b9/>
- The first appearance of an incident gout diagnostic code in the primary care record was deemed an incident gout diagnosis, assuming the patient had been registered with a TPP practice in England for at least 12 months prior to this code. Patients with a ULT prescription more than 30 days prior to their index code were deemed not to be incident diagnoses. Similarly, patients with recorded hospital admission(s) and/or emergency department attendance(s) for gout flares more than 30 days before the index code were deemed not to be incident diagnoses.
- We defined a gout flare as any of the following (adapted from a previously published definition^1^): 1) presence of a non-index diagnostic code for gout flare; 2) non-index admission with a primary gout diagnostic code; 3) non-index emergency department attendance with a primary gout diagnostic code; or 4) any non-index gout diagnostic code and a prescription for a flare treatment (colchicine, NSAID and/or corticosteroid) on same day as that code. Flares that occurred within 14 days of one another were excluded, to prevent double counting of the same flare.
- We defined the presence of a comorbidity as a current or ever-recorded diagnostic code for that condition on or before the index diagnosis date (for the gout cohort) or 1 March 2019 (for the reference population). A comorbidity was assumed not to be present if diagnostic codes for that condition were absent from the medical record.
- For people with diabetes mellitus, the most recent HBA1c reading in the 2 years prior to the index IA diagnosis date was captured and categorised according to whether it was above or below 58 mmol/mol.
- Chronic kidney disease was defined as an eGFR <60 ml/min/1.73 m^2^ (calculated from the most recent creatinine reading using the CKD-EPI formula with no ethnicity) and/or the presence of a diagnostic code for end-stage renal failure.
- Obesity/being overweight was defined according to the most recent BMI reading, assuming this reading was within 10 years of the index diagnosis date and the person was aged ≥16 at the time of the reading.
- Individual codelists are available at <https://codelists.opensafely.org> including:

<https://www.opencodelists.org/codelist/opensafely/ethnicity/2020-04-27/>

<https://www.opencodelists.org/codelist/opensafely/smoking-clear/2020-04-29/>

<https://www.opencodelists.org/codelist/opensafely/chronic-cardiac-disease/2020-04-08/>

<https://www.opencodelists.org/codelist/opensafely/diabetes/47ac0884/>

<https://www.opencodelists.org/codelist/opensafely/hypertension/2020-04-28/>

<https://www.opencodelists.org/codelist/opensafely/chronic-respiratory-disease/2020-04-10/>

<https://www.opencodelists.org/codelist/opensafely/chronic-liver-disease/2020-06-02/>

<https://www.opencodelists.org/codelist/opensafely/stroke-updated/2020-06-02/>

<https://www.opencodelists.org/codelist/opensafely/haematological-cancer/2020-04-15/>

<https://www.opencodelists.org/codelist/opensafely/lung-cancer/2020-04-15/>

<https://www.opencodelists.org/codelist/opensafely/cancer-excluding-lung-and-haematological/2020-04-15/>

<https://www.opencodelists.org/codelist/opensafely/renal-replacement-therapy/2020-04-14/>

## **Information Governance Statement**

NHS England is the data controller for OpenSAFELY-TPP; TPP is the data processor; all study authors using OpenSAFELY have the approval of NHS England. This implementation of OpenSAFELY is hosted within the TPP environment which is accredited to the ISO 27001 information security standard and is NHS IG Toolkit compliant. ^2^ Patient data has been pseudonymised for analysis and linkage using industry standard cryptographic hashing techniques; all pseudonymised datasets transmitted for linkage onto OpenSAFELY are encrypted; access to the platform is via a virtual private network (VPN) connection, restricted to a small group of researchers; the researchers hold contracts with NHS England and only access the platform to initiate database queries and statistical models; all database activity is logged; only aggregate statistical outputs leave the platform environment following best practice for anonymisation of results such as statistical disclosure control for low cell counts. ^3^ The OpenSAFELY research platform adheres to the obligations of the UK General Data Protection Regulation (GDPR) and the Data Protection Act 2018. In March 2020, the Secretary of State for Health and Social Care used powers under the UK Health Service (Control of Patient Information) Regulations 2002 (COPI) to require organisations to process confidential patient information for the purposes of protecting public health, providing healthcare services to the public and monitoring and managing the COVID-19 outbreak and incidents of exposure; this sets aside the requirement for patient consent. ^4^ This was extended in November 2022 for the NHS England OpenSAFELY COVID-19 research platform. ^5^ In some cases of data sharing, the common law duty of confidence is met using, for example, patient consent or support from the Health Research Authority Confidentiality Advisory Group. ^6^ Taken together, these provide the legal bases to link patient datasets on the OpenSAFELY platform. GP practices, from which the primary care data are obtained, are required to share relevant health information to support the public health response to the pandemic, and have been informed of the OpenSAFELY analytics platform. This study was supported by Dr Joanna Ledingham (clinical director for the National Early Inflammatory Arthritis Audit) as senior sponsor.

## **References**

1. Cipolletta E, Tata LJ, Nakafero G, Avery AJ, Mamas MA, Abhishek A. Association Between Gout Flare and Subsequent Cardiovascular Events Among Patients With Gout. *JAMA* 2022; **328**(5): 440-50.

2. NHS Digital. Data Security and Protection Toolkit. 2022. https://digital.nhs.uk/data-and-information/looking-after-information/data-security-and-information-governance/data-security-and-protection-toolkit (accessed July 7, 2023).

3. NHS Digital. ISB1523: Anonymisation Standard for Publishing Health and Social Care Data. 2022. https://digital.nhs.uk/data-and-information/information-standards/information-standards-and-data-collections-including-extractions/publications-and-notifications/standards-and-collections/isb1523-anonymisation-standard-for-publishing-health-and-social-care-data (accessed July 7, 2023).

4. UK Government. COVID-19: notification to GPs and NHS England to share information. 2022. https://web.archive.org/web/20200421171727/https://www.gov.uk/government/publications/covid-19-notification-to-gps-and-nhs-england-to-share-information (accessed July 7, 2023).

5. UK Government. Coronavirus (COVID-19): notice under Regulation 3(4) of the Health Service (Control of Patient Information) Regulations 2002. 2022. https://www.gov.uk/government/publications/coronavirus-covid-19-notification-to-organisations-to-share-information/coronavirus-covid-19-notice-under-regulation-34-of-the-health-service-control-of-patient-information-regulations-2002 (accessed July 7, 2023).

6. Health Research Authority. Confidentiality Advisory Group. 2023. https://www.hra.nhs.uk/about-us/committees-and-services/confidentiality-advisory-group/ (accessed July 7, 2023).
